# Cryogenic Aerosol Generation: Airborne Mist Particles Surrounding Liquid Nitrogen

**DOI:** 10.3390/ijerph17031071

**Published:** 2020-02-07

**Authors:** Byung Uk Lee

**Affiliations:** Aerosol and Bioengineering Laboratory, College of Engineering, Konkuk University, 120 Neungdong-ro, Gwangjin-gu, Seoul 05029, Korea; leebu@konkuk.ac.kr

**Keywords:** liquid nitrogen, cryogenics, aerosol generation, low temperature energy, air quality

## Abstract

Aerosol mist particles generated near the surface of a liquid nitrogen container were measured and analyzed. The particles present at various distances from the boiling surface of liquid nitrogen were detected using an optical particle counter. In this experiment, 3 micrometer particles exhibited a more than 100-fold increase in concentration due to the liquid nitrogen surface. However, 0.3 micrometer and 10 micrometer particles showed smaller variations (2% to 79%) in their concentrations in the vicinity of liquid nitrogen. The distance from the boiling surface of the liquid nitrogen strongly affected the variations in particle concentration. The variations in aerosol concentrations were significant within 20 cm of the liquid nitrogen surface. These results can be considered as a useful quantitative environmental guideline in cryogenic studies that use liquid nitrogen, and this concept can be applied to cryogenic aerosol mist generation mechanisms.

## 1. Introduction

Aerosol generation mechanisms have been researched and used in various aerosol studies [1,2,3,4]. Liquid nebulizers, air feeders, and spark generators have been developed for submicron particles, coarse particles, and nanoparticles, respectively, for aerosol experimental purposes [5,6,7]. Pressurized liquid sprays and electrosprays have also been used to generate fine mist particles [8,9]. Mixing gases and combustion have been considered as secondary particle generation methods for a gas to particle conversion mechanism and an incomplete oxidation byproduct particle production, respectively [10]. In this study, a novel concept of environmental aerosol generation is introduced and tested.

Liquid nitrogen (LN2) is widely used for the refrigeration of various engineering systems (for example, a magnetic resonance imaging (MRI) device) and the preservation of biological materials [11]. It is also used for studies on superfluids (liquid helium) and superconductors. At a pressure of 1 atmosphere (1atm = 1.104 bar), liquid nitrogen starts boiling at 77 K (−196.15 °C) and generates cold conditions in the air of the surrounding environment. In previous aerosol studies, liquid nitrogen has been used to maintain temperature gradient conditions in a low temperature range for thermophoresis [12].

In this study, aerosol particle fumes generated in the vicinity of liquid nitrogen were investigated using aerosol experimental methods. The particle fumes from the liquid nitrogen were analyzed in terms of their aerosol particle sizes and concentrations with temperature and humidity values. In this experiment, the distance of sampling locations from the boiling surface of liquid nitrogen was considered a parameter that affects the fumes from the liquid nitrogen. The diameters of the measured aerosol mist particles ranged from 0.3 to 10 μm. These particles were estimated to be affected by the condensation near the surface of liquid nitrogen. The size distributions and concentrations of aerosol particles were analyzed in terms of the ratio of the various mist-generated concentrations to the ordinary aerosol concentration. The experimental results showed that the concentrations of aerosol mist particles were strongly affected by the liquid nitrogen up to a distance of 20 cm from the liquid nitrogen surface, whereas they returned to their ordinary levels at distances greater than 30 cm from the liquid nitrogen surface. These experiments demonstrate that liquid nitrogen environments can be regarded as a method of mist particle generation for energy and environmental studies. In addition, the generated particles in the vicinity of a liquid nitrogen container can be used for studying air quality control devices for public health issues.

## 2. Methods

**Figure 1:**
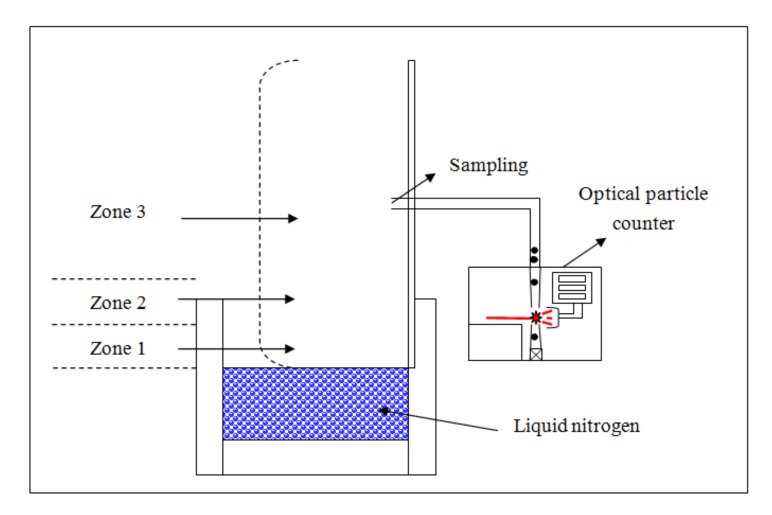
Schematic diagram of the experimental setup.

Figure 1 shows the schematic diagram of the experimental setup. The chamber containing liquid nitrogen was designed and fabricated. Inside the chamber (20 × 12 × 5 cm), 1.2 liters of liquid nitrogen were located and boiled at 77 K. The aerosol particles were sampled at distances of 10, 20, 30, 40, and 50 cm, and 1 m above the liquid nitrogen surface. The particle sizes and concentrations were measured using an optical particle counter (OPC; Portable particle counter, Model 3910, KANOMAX, Andover Township, NJ, USA). In the OPC, aerosol particles passed through a device in which the scattering signals between sampled particles and laser radiation light were translated into data for aerosol sizes and concentrations. The sampling flow rate was 28.3 L/min and the sampling time was 30 s per sample. The optical particle counter had six channels of sizes: 0.3 μm (0.3 ≤ D < 0.5 μm), 0.5 μm (0.5 ≤ D < 1 μm), 1 μm (1 ≤ D < 3 μm), 3 μm (3 ≤ D < 5 μm), 5 μm (5 ≤ D < 10 μm), and 10 μm (10 μm ≤ D). In addition, the temperature and relative humidity values were measured at the individual locations using a hygrometer (Testo 625, Germany). The laboratory environment where the experiment took place was air-conditioned, controlled (closed), and 6.9 m wide, 10.1 m long, and 2.7 m high. This environment was large enough to not be completely influenced by the liquid nitrogen. During the analysis, experimental results were converted into the ratio of aerosol mist concentrations to the ordinary (without LN_2_) indoor aerosol concentrations of the experimental environments, which is a particle concentration ratio, as expressed below:

(1) $$Particleconcentration\;ratio=\frac{Particleconcentration\;with\;LN_{2}}{Particleconcentration\;without\;LN_{2}}$$

For each individual condition, experiments were repeated at least three times in this study.

## 3. Results and Discussion

Table 1 lists the temperature and relative humidity values of the experimental locations. It can be observed that the temperature and humidity values show slight variations (ΔT: 0.6 °C; ΔRH: 5.4%) in the vicinity of LN_2_.

**Figure 2:**
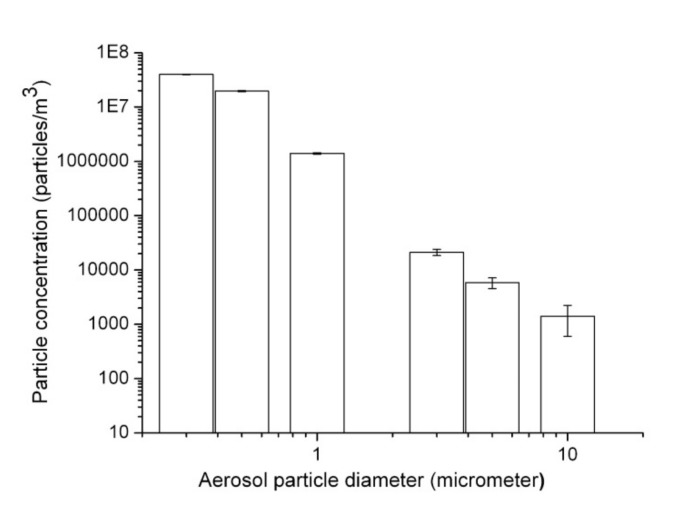
Aerosol particle size distributions in the experimental air environments (without LN_2_).

Figure 2 shows the concentrations of aerosol particles in the ordinary air environments of the experimental room without LN_2_. The concentrations of aerosol particles ranged from 10^7^ to 10^4^ particles/m^3^ for the particles, with sizes ranging from 0.3 to 10 μm. The replicative measurements showed that the fluctuation (standard deviations) in the particle concentrations was less than 58% of the average concentration.

**Figure 3:**
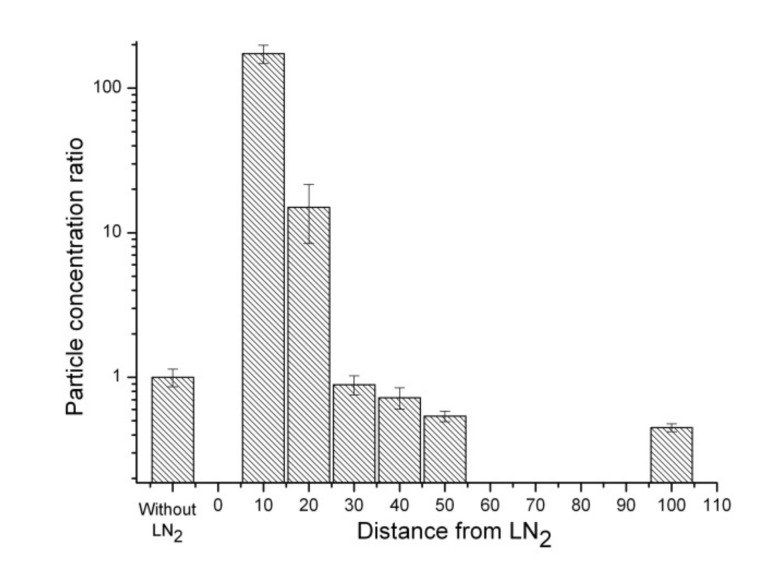
Concentration ratios of particles of size 3 μm at distances of 10, 20, 30, 40, 50, and 100 cm from the LN_2_ surface.

Table 2 presents the concentrations and optical sizes of aerosol particles with variations in distances from the LN_2_ boiling surface. Figure 3 shows the particle concentration ratios (the ratios of particle concentrations in the vicinity of liquid nitrogen to the ordinary particle concentrations) of 3 μm particles with respect to the distance from the LN_2_ surface. For the 3 μm particles, the concentration 10 cm above the LN_2_ surface increased from 2.1 × 10^4^ particles/m^3^ without LN_2_ (ratio = 1) to 3.68 × 10^6^ particles/m^3^ (ratio = 173), indicating an increase of > 100 times with statistical significance (*t*-test *p*-value < 0.05). At distances of 20 cm and 30 cm from the LN_2_ surface, the concentrations were 3.20 × 10^5^ particles/m^3^ (ratio = 15) (*t*-test *p*-value < 0.05) and 1.89 × 10^4^ particles/m^3^ (ratio = 0.89) (*t*-test *p*-value > 0.05), respectively. At distances greater than 30 cm from the LN_2_ surface, the concentration of these particles became similar (45% to 89%) to the ordinary indoor aerosol concentration without LN_2_.

**Figure 4:**
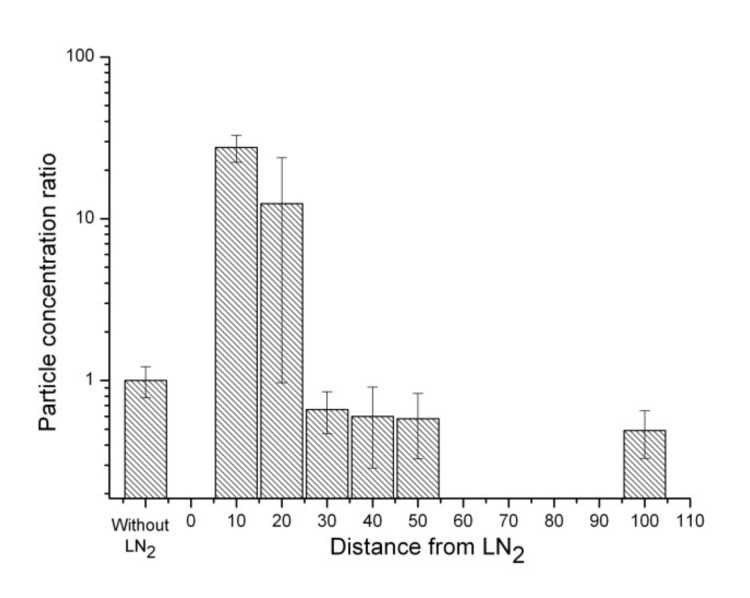
Concentration ratios of particles of size 5 μm at distances of 10, 20, 30, 40, 50, and 100 cm from the LN_2_ surfaces.

**Figure 5:**
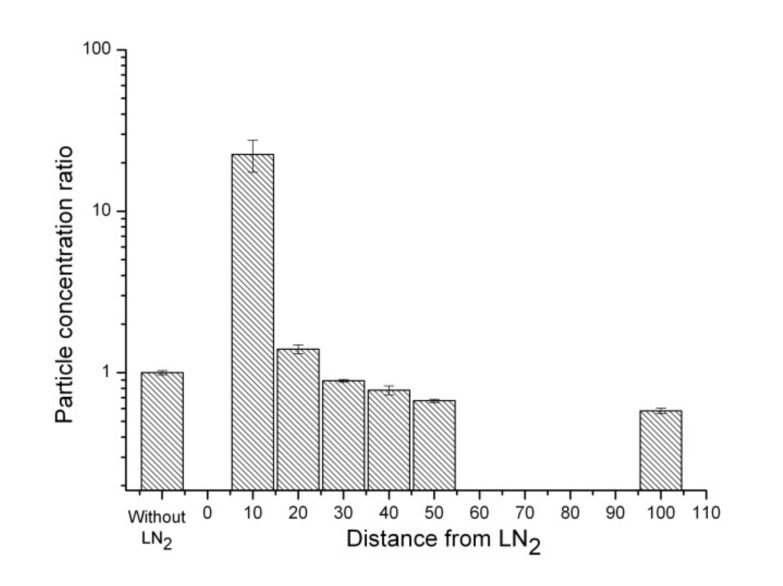
Concentration ratios of particles of size 1 μm at distances of 10, 20, 30, 40, 50, and 100 cm from the LN_2_ surfaces.

Figure 4 and Figure 5 show the concentration ratios for particles of sizes 5 and 1 μm, respectively, showing the distances from the LN_2_ surface. The concentrations of 5 μm particles were 1.63 × 10^5^ particles/m^3^ (ratio = 27.6) (*t*-test *p*-value < 0.05), 7.30×10^4^ particles/m^3^ (ratio = 12.4) (*t*-test *p*-value > 0.05), and 3.86 × 10^3^ particles/m^3^ (ratio = 0.66) (*t*-test *p*-value > 0.05) at distances of 10 cm, 20 cm, and 30 cm above LN_2_ surface, respectively. The concentrations of particle size 1 μm were 3.15 × 10^7^ particles/m^3^ (ratio = 22.5) (*t*-test *p*-value < 0.05), 2.00 × 10^6^ particles/m^3^ (ratio = 1.4) (*t*-test *p*-value < 0.05), and 1.25 × 10^6^ particles/m^3^ (ratio = 0.89) (*t*-test *p*-value < 0.05) at distances of 10 cm, 20 cm, and 30 cm above the LN_2_ surface, respectively. These particles (3 μm, 5 μm, 1 μm) showed an extreme increase in their concentrations from 173 times (particles of size 3 μm) to 22.5 times (particles of size 1 μm) more particles than the ordinary indoor conditions without LN_2_ boiling at 10 cm above the LN_2_ surface.

However, the submicrometer-sized particles of 0.3 and 0.5 μm showed different trends, as shown in Table 2. At 10 cm above the LN_2_ surface, the concentrations of submicrometer-sized particles became 21% (79% decrease) (*t*-test *p*-value < 0.05) and 43% (57% decrease) (*t*-test *p*-value < 0.05), respectively, of their ordinary indoor concentrations with statistical significance. The concentrations of these particles decreased, whereas the concentrations of particles of sizes 1, 3, and 5 μm increased in the vicinity of 10 cm of the LN_2_ boiling surface. The decrease in the concentrations of submicrometer-sized particles became insignificant at a distance of more than 20 cm above the LN_2_ surface; for example, the concentrations of 0.3 and 0.5 μm particles recovered by 98% (*t*-test *p*-value > 0.05) and 94% (*t*-test *p*-value < 0.05) of their ordinary indoor concentrations at the distance of 20 cm, respectively.

Throughout the experiment, variations in the concentrations of particles decreased as the distance from the LN_2_ surface increased. At the distances of 30 cm, 40 cm, 50 cm, and 1 m from the LN_2_ surface, the concentrations of 3 μm particles, which were highly affected by LN_2_, showed similar concentration levels to the surrounding ordinary aerosol concentrations (45% to 89% of the ordinary concentrations). Above 30 cm from the LN_2_ surface, other aerosol particles showed similar concentrations (42% to 103% of ordinary concentrations) to the fluctuated indoor particle concentrations without LN_2_.

**Figure 6:**
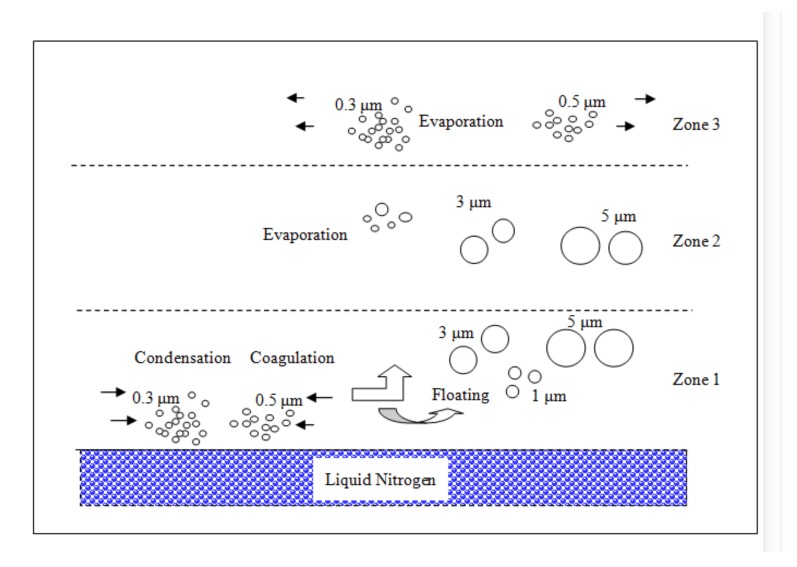
Model of transition of aerosol particles in the vicinity of the LN_2_ surfaces.

These experimental results can be explained by dividing the nearby space of LN_2_ in the following way. The zones of the LN_2_ were divided into three groups: within 10 cm (zone 1), 10 to 20 cm (zone 2), and more than 30 cm (zone 3) environments from the LN_2_ boiling surface, as shown in Figure 1. Figure 6 shows a model of transition of the aerosol particles in the zones.

In the zone 1 environment (10 cm from the LN_2_ surface), the variation in the particle concentrations can be explained by the fact that submicron particles present on the surface transformed into particles of sizes ranging from 1 to 5 μm due to the condensation of surrounding gases on the submicron particles. Then, the grown aerosol particles floated and spread near the LN_2_ surface environments (Figure 6). As shown in Table 1, the decreased concentrations of particles of sizes 0.3 and 0.5 μm are −3.2 × 10^7^ and −1.1 × 10^7^ particles/m^3^, respectively. The increased concentrations of particles of sizes 1, 3, and 5 μm are 3.0 × 10^7^, 3.7 × 10^6^, and 1.6 × 10^5^ particles/m^3^. This result can be interpreted as particles of sizes 0.3 and 0.5 μm (4.3 × 10^7^ particles/m^3^) transform into particles of sizes 1, 3, and 5 μm (3.4 × 10^7^ particles/m^3^) because of the condensation and possible merging of particles caused by LN_2_ (Figure 6).

In zone 2 (10 cm to 20 cm from the LN_2_ surface), the floated particles of sizes ranging from 3 to 5 μm maintained their shapes. However, particles of other sizes, such as 1 μm and submicrometer-sized particles, returned to their original shape. This resulted in decreased aerosol concentrations of the 1 μm particles present at this location. This change was estimated to be a result of the evaporation of condensed materials present on the surfaces of 1 μm particles (Figure 6).

In the zone 3 environment (30 cm from the LN_2_ surface), the concentration of aerosol particles approached the ordinary fluctuating concentrations of the surrounding experimental environments without LN_2_. The floating mist particles could not be influenced over 30 cm from the LN_2_ surface under this experimental condition.

The general equation for the concentration of the aerosol particles could be expressed by the following differential form:

(2) $$\frac{\partial n_{k}}{\partial t}+\nabla \cdot n_{k}\vec{V}={\left(\nabla \cdot D\nabla n_{k}\right)}_{diffusion}+{\left[\frac{\partial n_{k}}{\partial t}\right]}_{growth}-{\left[\frac{\partial n_{k}}{\partial t}\right]}_{evaporation}+{\left[\frac{\partial n_{k}}{\partial t}\right]}_{coagulation}$$

Equation (2) is a differential equation of specific-sized particle concentrations, “n_k_”. For 0.3 and 0.5 μm particles, condensational growth ${\left[\frac{\partial n_{k}}{\partial t}\right]}_{growth}$ and coagulations ${\left[\frac{\partial n_{k}}{\partial t}\right]}_{coagulation}$ transformed them (decease of concentration) into 1, 3, and 5 μm particles (increase of concentration) in zone 1 of Figure 6. However, 1 μm particles experienced evaporation $-{\left[\frac{\partial n_{k}}{\partial t}\right]}_{evaporation}$ in zone 2. Therefore, the concentrations of 0.3 μm particles and 0.5 μm particles increased, while that of 1 μm particles decreased in zone 2. The condensation, coagulation, and evaporation occurred in zones 1 and 2. However, the floating aerosol mist particles from the LN_2_ surface could not approach the distant zone 3.

For the generation of aerosol particles, it can be considered that more than ten times the number of particles with diameters ranging from 1 to 5 μm can be artificially generated using the LN_2_ surface, and this can be treated as a cryogenic aerosol generator for these particles. For the generation of 3 and 5 μm mist particles, we can use the environments 10 and 20 cm (zone 1 and zone 2) from the LN_2_ surface. For the generation of 1 μm aerosol mist particles, we can use the environment 10 cm (zone 1) from the LN_2_ surface. Therefore, the environments of the LN_2_ surface can be used for the momentary generation of aerosol mist particles, of sizes ranging from 1 to 5 μm.

These experimental results can have several potential applications. First, it can be recognized that aerosol mist particles of sizes 1, 3, and 5 μm are generated in surrounding environments when LN_2_ is used in working places, such as the preservation process for biological materials and the cooling process for superconducting devices. Any particle-sensitive device can be affected in a precautionary working place with LN_2_. Second, the generated particles from LN_2_ can be used for testing control devices targeting smog mist particles. There are demands for the development of control devices against smog aerosol mist particles which are hazardous to human health [13,14]. The mist particles generated from LN_2_ can be used as test aerosols for a high-concentration test of the control devices with easy treatments after experiments due to its short residence time. Further new applications can be developed using this newly found cryogenic aerosol generation mechanism. There are limitations to this study. Experiments with various environmental conditions, such as very dry or humid situations, can be conducted with more controlled fluctuations in aerosol particle concentrations in the experimental chamber. Measurements via other devices, such as an aerodynamic particle spectrometer, which has more size channels, and other nanoparticle detection devices, can be considered in future studies.

## 4. Conclusions

In this study, aerosol mist particles of sizes ranging from 1 to 5 μm were generated in the vicinity of 10 to 20 cm from the LN_2_ boiling surface. In particular, the 3 μm particles were strongly affected by the LN_2_ environments. These experimental results are estimated due to the condensation, evaporation, and floating of particles in the vicinity of the LN_2_ surface. Different zones of the LN_2_ surface can be used as cryogenic aerosol generating spaces for aerosol studies. However, there are limitations to this study. For example, the number of division of sampling distances from the LN_2_ boiling surface to the outside could be considered. In addition, further studies need to be conducted for a lifetime study of the generated mist particles. This experimental result can provide useful information on the applications of liquid nitrogen.

## Figures and Tables

**Table 1:** Temperature and relative humidity values in the vicinity of liquid nitrogen (LN_2_).

	Temperature (°C)	Relative Humidity (%)
Without LN_2_	25.2 ± 0	64.9 ± 0.1
10 cm from LN_2_	25.8 ± 0.1	62.1 ± 0.2
20 cm from LN_2_	25.8 ± 0.1	63.8 ± 0.1
30 cm from LN_2_	25.5 ± 0	64.6 ± 0.1
40 cm from LN_2_	25.3 ± 0	67.5 ± 0.1
50 cm from LN_2_	25.3 ± 0	66.8 ± 0.1
100 cm from LN_2_	25.2 ± 0	64.9 ± 0.1

**Table 2:** Aerosol particle concentrations (particles/m^3^) in the vicinity of liquid nitrogen (LN_2_).

	0.3 μm	0.5 μm	1 μm	3 μm	5 μm	10 μm
Without LN_2_	4.0 × 10^7^ ± 3.0 × 10^5^	2.0 × 10^7^ ± 4.0 × 10^5^	1.4 × 10^6^ ± 4.4 × 10^4^	2.1 × 10^4^ ± 2.9 × 10^3^	5.9 × 10^3^ ± 1.3 × 10^3^	1.4 × 10^3^ ± 8.1 × 10^2^
10 cm from LN_2_	8.4 × 10^6^ ± 3.6 × 10^6^	8.4 × 10^6^ ± 1.1 × 10^6^	3.2 × 10^7^ ± 7.0 × 10^6^	3.7 × 10^6^ ± 5.3 × 10^5^	1.6 × 10^5^ ± 3.1 × 10^4^	3.3 × 10^2^ ± 8.1 × 10^1^
20 cm from LN_2_	3.9 × 10^7^ ± 6.4 × 10^5^	1.8 × 10^7^ ± 8.2 × 10^5^	2.0 × 10^6^ ± 1.3 × 10^5^	3.2 × 10^5^ ± 1.4 × 10^5^	7.3 × 10^4^ ± 6.7 × 10^4^	8.2 × 10^2^ ± 5.1 × 10^2^
30 cm from LN_2_	4.1 × 10^7^ ± 2.6 × 10^5^	1.8 × 10^7^ ± 6.5 × 10^5^	1.2 × 10^6^ ± 2.6 × 10^4^	1.9 × 10^4^ ± 2.8 × 10^3^	3.9 × 10^3^ ± 1.1 × 10^3^	6.8 × 10^2^ ± 2.9 × 10^2^
40 cm from LN_2_	4.2 × 10^7^ ± 3.3 × 10^5^	1.6 × 10^7^ ± 7.4 × 10^5^	1.1 × 10^6^ ± 6.9 × 10^4^	1.5 × 10^4^ ± 2.6 × 10^3^	3.5 × 10^3^ ± 1.8 × 10^3^	1.4 × 10^3^ ± 1.0 × 10^3^
50 cm from LN_2_	4.2 × 10^7^ ± 6.0 × 10^4^	1.4 × 10^7^ ± 3.3 × 10^5^	9.4 × 10^5^ ± 2.1 × 10^4^	1.1 × 10^4^ ± 9.3 × 10^2^	3.4 × 10^3^ ± 1.5 × 10^3^	1.1 × 10^3^ ± 3.2 × 10^2^
100 cm from LN_2_	4.2 × 10^7^ ± 1.1 × 10^5^	1.2 × 10^7^ ± 2.3 × 10^5^	8.1 × 10^5^ ± 2.9 × 10^4^	9.6 × 10^3^ ± 6.0 × 10^2^	2.9 × 10^3^ ± 9.4 × 10^2^	5.9 × 10^2^ ± 1.1 × 10^2^
